# Free Amino Acids Profile and Expression Analysis of Core Genes Involved in Branched-Chain Amino Acids Metabolism during Fruit Development of Longan (*Dimocarpus longan* Lour.) Cultivars with Different Aroma Types

**DOI:** 10.3390/biology10080807

**Published:** 2021-08-20

**Authors:** Wenshun Hu, Baiyu Wang, Muhammad Moaaz Ali, Xiuping Chen, Jisen Zhang, Shaoquan Zheng, Faxing Chen

**Affiliations:** 1 College of Horticulture, Fujian Agriculture and Forestry University, Fuzhou 350002, China; huwenshun06@163.com (W.H.); muhammadmoaazali@yahoo.com (M.M.A.); 2 Fujian Breeding Engineering Technology Research Center for Longan & Loquat, Fruit Research Institute, Fujian Academy of Agricultural Sciences, Fuzhou 350013, China; cxp2516@126.com; 3 Center for Genomics and Biotechnology, Haixia Institute of Science and Technology, College of Life Sciences, Fujian Agriculture and Forestry University, Fuzhou 350002, China; wbycyx@163.com (B.W.); zjisen@126.com (J.Z.)

**Keywords:** fruit quality, L-alanine, L-leucine, BCAT, IPMS, IPMD

## Abstract

**Simple Summary:**

In this study, three longan cultivars, including non-aroma types ‘Shixia’ (SX), ‘Lidongben’ (LDB), and strong aroma type ‘Xiangcui’ (XC), were selected to analyze free amino acids (FAAs) variations at six distinct growth stages. The genome-wide identification and expression analysis of genes related to the branched-chain amino acids (BCAA) synthesis pathway were carried out. Results showed that thirty-six FAAs were identified, which increased drastically with fruit development until ripening. During the period of rapid fruit expansion, the aroma of XC changed from light to strong, and the contents of L-alanine and L-leucine were significantly higher than those of SX and LDB. The content of Leu was negatively correlated with the expression of *DilBCAT*1, -6, and -9 in three varieties, but positively correlated with *DilBCAT*16, indicating that these four genes may be responsible for the different synthesis and degradation of Leu among cultivars.

**Abstract:**

Amino acids are important component of fruit nutrition and quality. In this study, three longan cultivars, including non-aroma types ‘Shixia’ (SX), ‘Lidongben’ (LDB), and strong aroma type ‘Xiangcui’ (XC), were selected to analyze free amino acids (FAAs) variations at six distinct growth stages (S1–S6). The genome-wide identification and expression analysis of genes related to the branched-chain amino acids (BCAA) synthesis pathway were carried out. Results showed that 36 FAAs were identified, and the total FAAs content ranged from 2601.0 to 9073.5 mg/kg, which increased drastically with fruit development until ripening. L-glutamic acid (Glu), L-alanine (Ala), L-arginine (Arg), γ-Aminobutyric acid (GABA), L-aspartic acid (Asp), L-leucine (Leu), hydroxyl-proline (Hypro), and L-serine (Ser) were the predominant FAAs (1619.9–7213.9 mg/kg) in pulp, accounting for 62.28–92.05% of the total amino acids. During the period of rapid fruit expansion (S2–S4), the aroma of XC changed from light to strong, and the contents of L-alanine (Ala) and L-leucine (Leu) were significantly higher than those of SX and LDB. Furthermore, a total of two 2-isopropyl malate synthase (IPMS), two 3-isopropyl malate dehydrogenase (IPMD), and 16 BCAA transferase (BCAT) genes were identified. The expression levels of *DilBCAT*1, -6, and -9 genes in XC were significantly higher than those in SX and LDB, while *DilBCAT*16 in XC was lower. The content of Leu was negatively correlated with the expression of *DilBCAT*1, -6, and -9 in three varieties, but positively correlated with *DilBCAT*16, indicating that these four genes may be responsible for the different synthesis and degradation of Leu among cultivars.

## 1. Introduction

Longan (*Dimocarpus longan* Lour.) is an economically important crop in tropical and subtropical regions of Southeast Asia i.e., China, Thailand, Vietnam, etc. Longan possesses numerous nutritional and functional components, including polysaccharides [1], amino acids [2,3], polyphenols and alkaloids [4,5], flavonoids, lipids [6], organic acids, vitamin C, minerals [7,8], and volatile compounds [9]. Amino acids exist in both bound and free forms in plants. The bound amino acids of longan are not hydrolyzed immediately during the course of eating and have little contribution to the flavor. Each FAA has its own taste–as one of or a combination of sweet, bitter, umami, salty, and sour [10] which directly or indirectly affects the taste and even aroma of longan fruit. Therefore, the component identification and dynamic change analysis of FAAs in different longan varieties during ripening can help understand the essence of the formation of flavor quality.

BCAAs (Leu, Ile, Val), aromatic amino acids (Phe, Tyr, Trp), sulfuric amino acids (Cys, Met), Ala, and Asp are the main amino acid substrates for aroma compounds synthesis [2,3,10,11,12]. Among them, BCAAs are the important precursors for the synthesis of branched-chain esters in fruits, which has been reported in apple [13], banana [14,15], melon [16], and other horticultural crops. Ala, Glu, and Thr provide important prerequisites for the synthesis of BCAAs, and BCAAs can be converted into their corresponding α-keto acids by BCAT, and further metabolized into branched-chain aldehydes, alcohols, and esters by a series of enzymes. The IPMS and IPMD catalyze the committed step of Leu biosynthesis [17,18]. Genes encoding the key enzymes involved in the BCAA metabolic pathway have been cloned and studied in several plants, but have still not been reported in longan.

**Figure 1:**
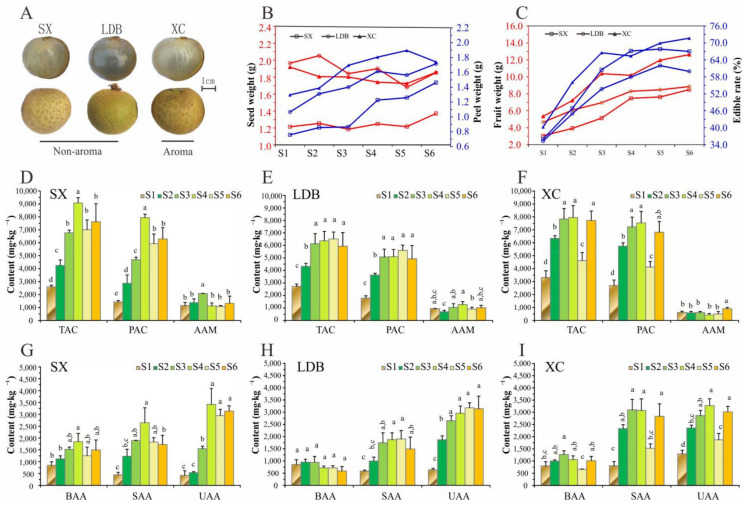
Changes in phenotypic traits and FAAs at six stages of fruit development of three longan varieties (S1–S6). (**A**) The mature fruits of three varieties with typical flavor. (**B**,**C**) Changes in main fruit characters. (**D**–**F**) Changes in amino acid composition in SX, LDB, and XC, respectively. (**G**–**I**) Changes in amino acids categorized with respect to taste in SX, LDB, and XC, respectively. TAC: total amino acid content, PAC: protein amino acids, AAM: amino acid metabolites; BAA: bitter amino acid, SAA: sweet amino acid, UAA: umami amino acid. Values represent the mean of three biological samples, and different uncapitalized letters indicate an extremely significant difference between the different stages (LSD test, *p* ≤ 0.01).

The existing research mainly focuses on the FAAs components and content of longan fruit at maturity [19,20]. Although Huang et al. [21] reported the FAAS content (seventeen common protein amino acids and γ-aminobutyric acid) in the middle and late stage of LDB fruit development, all these test varieties have no aroma. Our previous results showed that the content of FAAs in 80 longan fruits of different genotypes were significantly different [22], in which the Leu content of XC was 2.1 times higher than the average (the second highest), while the Leu content of SX and LDB was lower than the average, and the total content of BCAAs in XC was about 2 times of that in SX and LDB. However, the dynamics of FAAs contents and related gene expression during fruit development of different variety longan are still unclear. In the present work, these three varieties (Figure 1A) with different aroma characteristics were selected to analyze the accumulation rule of FAAs (including protein amino acids and their derivatives) and the expression profiles of core genes involved in BCAAs metabolism during fruit ripening. The findings can help to understand the essence of fruit flavor formation by FAAs and provide scientific guidance for the further breeding of new longan cultivars.

## 2. Materials and Methods

### 2.1. Plant Materials

The fruits from three longan cultivars were collected from an orchard located at the Institute of Fruit Science, Fujian Academy of Agricultural Sciences (119°20′ E, 26°7′ N, altitude 24 m). The longan cultivars were ‘Shixia’ (SX, early-maturing cultivar in China, medium fruit size, strong sweet and crisp aril), ‘Lidongben’ (LDB, late-maturing cultivar in China, medium fruit size, strong sweet, juicy, and tender aril), and ‘Xiangcui’ (XC, late-maturing cultivar of the bud sport variety of ‘Biew Kiew’ from Thailand, with a large fruit size, as well as a fragrant, sweet and crisp aril).

Twenty fruits per replicate were harvested at six different developmental stages [100, 110, 120, 130, 135, and 140 days after pollination (DAP) from LDB and XC, and 80, 90, 100, 110, 115, and 120 DAP from SX]. Three biological replicates were sampled. The six different harvesting points were between July and September 2018, representing the six different maturation stages, namely, the seed coat color-changing stage (S1): the seed coat color changed from white to brown, the green pericarp stage (S2), the fruit expanding stage (S3), the fruit peel color-changing stage (S4): the pericarp lost green and turned yellow gradually, mature fruit stage (S5), and the full-mature fruit stage (S6). The evaluation of fruit phenotypic characters refers to Hu et al. [23]. The samples’ flesh was cut into small pieces, frozen in liquid nitrogen, and stored at −80 °C prior to further analysis.

### 2.2. Chemicals and Reagents

All chemicals and reagents used in this study were analytical or of HPLC grade. Amino acids mixture standard solutions i.e., Type AN-Ⅱ and Type B were purchased from FUJIFILM Wako Pure Chemical Corporation (Osaka, Japan). The amino acid standard solutions included 17 types of protein amino acids, with the following standard codes: Asp: L-aspartic acid, Thr: L-threonine, Ser: L-serine, Glu: L-glutamic acid, Gly: glycine, Ala: L-alanine, Cys: L-cystine, Val: L-valine, Met: L-methionine, Ile: L-isoleucine, Leu: L-leucine, Tyr: L-tyrosine, Phe: L-phenylalanine, His: L-histidine, Lys: L-lysine, Arg: L-arginine, Pro: L-proline, and 21 types of non-protein amino acids, with the following standard codes: P-Ser: phosphoserine, Tau: taurine, PEA: O-phosphosphorylethanolamine, urea, Sar: sarcosine, α-AAA: α-amino-adiic acid, Cit: L-citrulline, α-ABA: α-aminobutyric acid, Cystha: cystathionine, Nle: L-norleucine, H-cysteine: homocysteine, β-Ala: β-alanine, β-AiBA: β-aminoisobutyric acid, GABA: γ-Aminobutyric acid, 3Mehis: 3-methylhistidine, 1Mehis: 1-methylhistidine, Car: carnosine, Ans: anserine, Hylys: hydroxylysine, Orn: ornithine, NH_4_Cl, EtNH_2_: ethanolamine, Hypro: hydroxylproline. The volume of each amino acid was 100 nmol mL^−1^.

### 2.3. FAAs Determination

Sample preparation was carried out according to the method of Chen et al. [24] with some modifications. Briefly, the fruit aril was ground to a fine powder in the mortar with liquid nitrogen. The 3 g powder was mixed with 2 mL 60 mg mL^−1^ 5-sulfosalicylic acid in a 10 mL centrifuge tube, incubated in a water bath for 1 h at 37 °C. The 1 mL 0.06 mol·mL^−1^ HCl and 1 mL 10 mg mL^−1^ EDTA-2Na were added and mixed well. The homogenate was then centrifuged at 13,500 r min^−1^ for 15 min by Avanti J-26 XP centrifuge (Beckman Coulter, Brea, CA, USA). One milliliter of supernatant was mixed with 1 mL pH 2.2 citric acid-citrate sodium buffer, followed by filtration with a 0.45 μm millipore filter membrane.

The amino acid composition and content were measured by L-8900 amino acid automatic analyzer (Hitachi, Tokyo, Japan), with a 20 μL filtrate injection, a type of 855-350 ion exchange chromatographic column (4.6 mm × 60 mm, 3 μm), a column temperature of 134 °C, a detection time of 125 min, and monitored at the wavelength of 570 nm and 440 nm. Quantification was carried out using the calibration curves of the respective standards. Amino acid concentrations were expressed in mg·kg^−1^ flesh weight.

### 2.4. Gene Identification and Analysis

The genomic data of *Arabidopsis thaliana* and *Solanum lycopersicum* were obtained from Phytozome (https://phytozome.jgi.doe.gov, accessed on 7 February 2021). The IPMS, IPMD, and BCAT genes of tomato and Arabidopsis were retrieved from previous reports [25,26]. Based on the genome data of SX longan varieties generated by our laboratory (Submitted), two strategies were used to identify gene families. Firstly, Swissprot gene function annotation and keyword search were performed to obtain the sequence and annotation information of targeted genes. Secondly, protein blast verification was performed using the NCBI database (https://www.ncbi.nlm.nih.gov, accessed on 11 February 2021). In addition, the BCAT protein sequences of *Arabidopsis* and *Solanum* were used as queries to search against the litchi genome (unpublished) through the BLASTP program with an E-value cutoff of 1 × 10^−5^ and identity > 40%. The protein sequences were aligned using MUSCLE program in MEGA X [27] with default parameters. Then NJ (neighbor-joining) phylogenetic tree was constructed with the following parameters: the poisson model and pairwise deletion, as well as bootstrap for 1000 replicates. A total of 45 transcriptome sequencing databases (unpublished) of fruit development were obtained from the SX, LDB, and XC at five stages (S1, S2, S3, S4, and S6), including three biological repeats, and then the FPKM (fragments per kilobase per million) expression level of target genes was analyzed.

### 2.5. Statistical Analysis

Statistical analysis was performed using Microsoft Excel 2007, SPSS 25.0 (IBM Corporation, Armonk, NY, USA), and R version 3.6.1 (https://www.r-project.org/, accessed on 8 October 2020). The figures in this article were prepared with CorelDRAW X8 (Corel Corporation Ltd., Ottawa, ON, Canada). Comparisons were statistically evaluated by using one-way analysis of variance (ANOVA) followed by LSD’s multiple range test. Partial least squares-discriminant analysis (PLS-DA) was performed using ropls v1.18.8 in R [28]. The significantly accumulating metabolites with variable importance in projection (VIP) value ≥ 1.0 and *p*-value ≤ 0.05 were selected from LDB, XC and SX.

## 3. Results

### 3.1. Changes in Phenotypic Traits and FAAs during Fruit Development

The entire fruit developmental process was divided into 6 stages i.e., S1–S6. The fruit grows slowly in the early phase of development (early maturing variety SX was 0–80 days after female flower pollination, and late-maturing varieties LDB and XC were 0–100 days after pollination). S1 was the last stage of this period, the color of the seed coat changed from white to brown, the flesh was slightly bitter and astringent, with obvious grass flavor and no aroma. During the rapid fruit expansion phase (S2–S4), the fruit weight, peel weight, and the edible rate increased rapidly, but the seed weight remained stable (Figure 1B,C). During this period, the sweetness of SX and LDB was gradually obvious, and the sweetness and aroma of XC were gradually enhanced, and all three varieties were close to maturity at S4 stage. At the ripening phase (S5 and S6), SX and LDB had a strong sweet flavor, while XC fruit had a sweet flavor and a strong aroma.

While comparing with 38 amino acid standards, 36, 36, and 34 FAAs (Table S1) were identified from the pulp of SX, LDB, and XC, respectively. The Sar and Val were not detected in XC. The content changes of FAAs were also different among varieties during fruit development (Figure 1D–F). The total amino acid contents of SX, LDB, and XC were 2601.0~9073.5 mg/kg, 2727.4~6366.2 mg/kg, and 3309.9~7956.8 mg/kg, respectively. They all showed an increasing trend on the whole, reached the peak at S4 (LDB was at S5), and then remained stable, while SX and XC varieties decreased suddenly at S5 and increased at S6. The changing trend of protein amino acid content was the same as that of total amino acid, but the change of total metabolite content was small. The contents of 7 essential amino acids (Thr, Val, Met, Ile, Leu, Phe, Lys) accounted for 4.52–8.71%, 4.12–6.65%, and 4.78–8.74% of the total FAAs, respectively (Table S1).

The taste characteristics of FAAs mainly include the following three categories; bitter (Tau, Urea, Val, Met, Ile, Leu, Tyr, Phe, Lys, His, Arg), sweet (Thr, Ser, Gly, Ala, b-Ala, Hypro, Pro) and umami (Asp, Glu, Sar, Orn). As shown in Figure 1G–I and Table S1, the contents of tasty amino acids in the three varieties accounted for 65.88–87.11%, 76.30–88.94%, and 87.61–93.14%, respectively. Among them, Glu, Ala, Arg, Asp, Leu, Hypro, and Ser were the predominant FAAs. Above all, the synergistic effect of these tasty amino acids formed the characteristic flavor of the different cultivar fruit during the specific development period.

### 3.2. Multivariate Analysis of Identified FAAs

**Figure 2:**
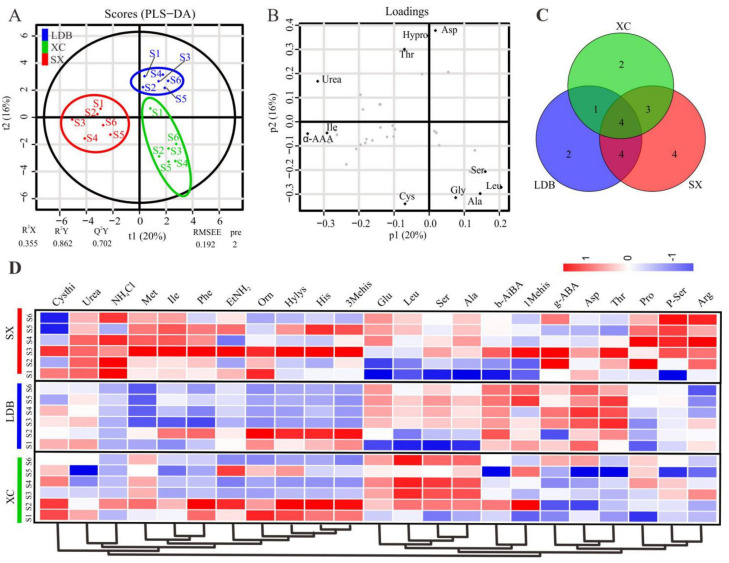
Multivariate analysis of FAAs identified in different longan cultivars during fruit development. (**A**) PLS-DA scores plot of FAAs profiles from SX, LDB, and XC fruits. (**B**) PLS-DA loadings plot for three cultivars. (**C**) Venn diagram of different significantly discriminant FAAs (content data were log-transformed and then normalized) from different stages. (**D**) Heatmap cluster showing the content of 23 FAAs in three longan cultivars.

FAA metabolites in the SX, LDB, and XC extracts recorded at six different fruit developmental and maturity stages were subjected to multivariate statistical analysis. PLS-DA, to explore the intrinsic differences among cultivars based on the aligned dataset. Samples of six stages were separated and classified into three distinct clusters by cultivars presented in the PLS-DA score plot. XC was separated from SX and LDB along t1 (20%) and t2 (16%). The quality parameters for the model were verified with R^2^X = 0.355, R^2^Y = 0.862, and Q^2^Y = 0.702 (Figure 2A), and the permutation testing indicated no overfitting (Figure S1).

Moreover, the significantly discriminant FAAs variables that were distant from the origin were indicated in the corresponding loading plots (Figure 2B). Based on the PLS-DA model and one-way ANOVA, 23 significantly discriminant FAAs were screened out with VIP value ≥1.0 and *p* ≤ 0.05 (Table S1). Among them, 17, 12, and 13 FAAs were identified from SX, LDB, and XC, respectively. The 5 common differential FAAs (Ser, Glu, b-AiBA, Ala, Leu) were observed by taking an intersection, and each cultivar had its unique differential FAAs (Figure 2C). A heatmap cluster analysis was conducted to illustrate the content difference of these discriminant metabolites among three cultivars (Figure 2D), and the results showed that the aromatic genotype (XC) had a greater tendency to accumulate Glu, Leu, Ser, and Ala than non-aromatic genotypes (SX and LDB) during fruit development.

### 3.3. Identification and Expression Analysis of Gene Family Related to BCAAs Synthesis and Degradation during Fruit Development

**Figure 3:**
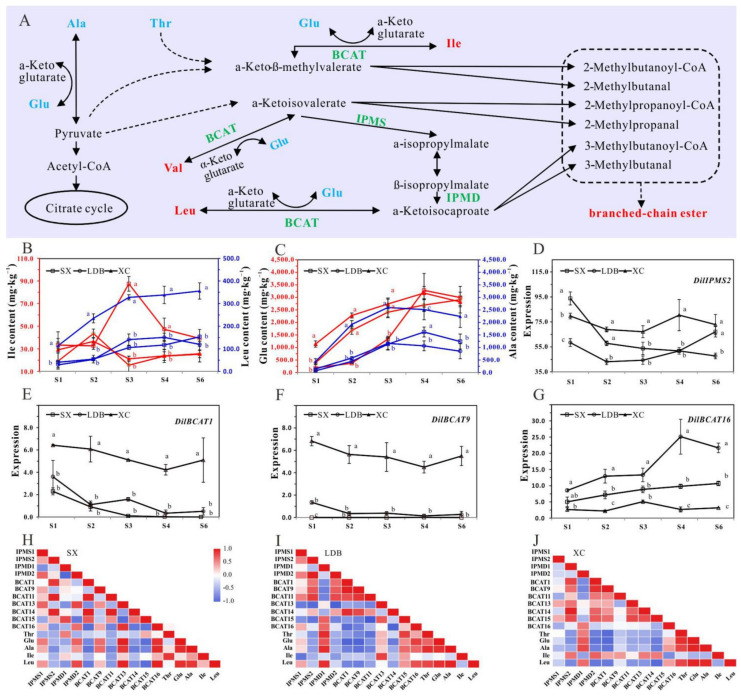
The BCAAs biosynthesis in longan fruit and expression patterns of genes encoding BCAAs biosynthesis and degradation during fruit development. (**A**) Pathway involved in BCCAs synthesis and degradation was adapted from Alsmairat et al. [14] and Wang et al. [31]. Dashed lines indicate multiple chemical reaction steps. (**B**,**C**) The main amino acids accumulated differently among varieties. (**D**–**G**) Transcription profiles of BCAAs biosynthesis-related genes in longan fruit. (**H**–**J**) Heatmap of Pearson’s correlation coefficient. Each data point represents the mean of three biological samples, and vertical bars represent ± standard error.

The BCAAs (including Val, Leu, and Ile) are primary metabolites synthesized in plants and are essential amino acids for the human body. These are synthesized from Thr or pyruvate in plastids [29,30], which have been well investigated (Figure 3A). Further data analysis showed that during the rapid fruit expansion phase (S2–S4), the contents of Leu and its biosynthesis-related Ala in aromatic variety XC were significantly higher than those in non-aromatic varieties (SX and LDB) at the same development stage (Figure 3B,C). Therefore, we carried out the identification and expression analysis of some genes in the biosynthesis (map00290) and degradation (map00280) pathways of BCAAs (http://www.kegg.jp/, accessed on 12 October 2020).

**Figure 4:**
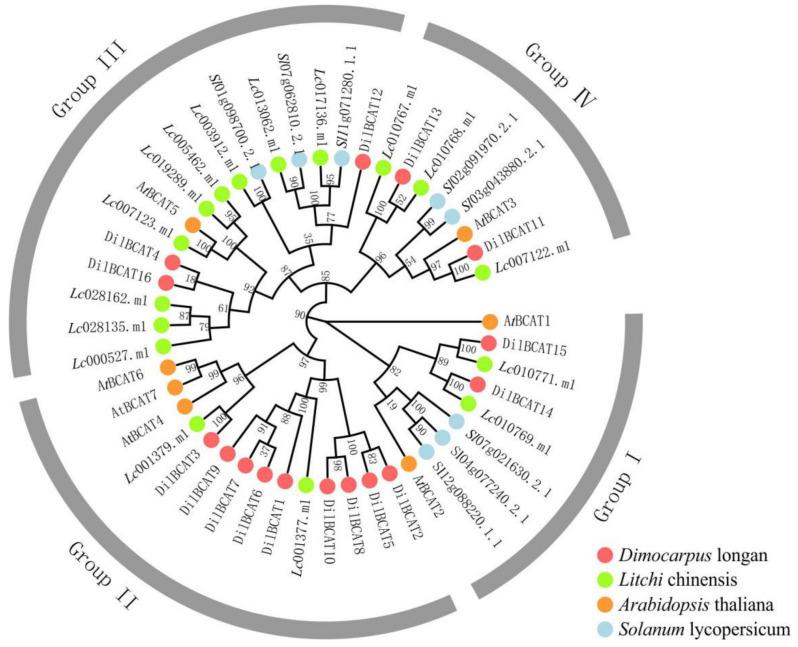
Phylogenetic tree of BCAT genes. The NJ tree was constructed by 47 BCAT proteins sequences from *longan* (16), *Litchi* (16), *Arabidopsis* (7), *Solanum* (8). The BCAT proteins of each genus were distinguished by different color dots.

Based on the gene function annotation and protein blast, 20 putative genes related to the metabolism and synthesis of BCAAs were identified in three families of longan genome, including 2 *DilIPMS*, 2 *DilIPMD*, and 16 *DilBCAT,* which were unevenly distributed on chromosomes 1, 5, 6, 7, 8, 9, 11, and 14, encode 609–825, 408–418, and 111–412 amino acids, respectively (Table S2). In addition, 16 BCAT genes were also identified from litchi genome (Table S3). The phylogeny of the BCAT family was analyzed by combining the previous reports on Arabidopsis [25] and tomato [26]. The results showed that the BCAT gene family could be divided into four subgroups i.e., group I, II, III, and IV. The BCAT family in longan and litchi of *Sapindaceae* were both obviously expanded, and *DilBCAT* genes had multiple adjacent copies on chromosomes 5 and 11 (Figure 4, Table S2).

The gene expression levels of *DilIPMS* and *DilIPMD* in three varieties were relatively high, with FPKM average value of about 16~70 (Table S2). Among the 16 *DilBCAT* genes, the expression levels of *DilBCAT*11 and *DilBCAT*15 were higher, and the average FPKM value was ~100, which basically decreased with the developmental process. In addition, the expression levels of *BCAT*2, -3, -4, -5, -7, -8, -10, and -12 genes were extremely low or undetectable (Table S2). During fruit development, the gene expression levels of *DilIPMS*2, *DilBCAT*1, *DilBCAT*6, and *DilBCAT*9 in the XC variety were relatively higher. Among them, *DilBCAT*1, -6, and -9 were significantly higher than those in SX and LDB varieties, and *DilBCAT*16 gene expression level of XC variety was significantly lower than those of SX and LDB varieties at S2, S4, and S6 (Figure 3D–G).

The correlation of the aforementioned 12 genes with a relatively high expression level and 36 kinds of FAAs was analyzed (Figure 3H–J, Tables S4–S6). The results showed that the content change of Glu had extremely significant correlation with Ala and Leu. Meanwhile, the Leu content was negatively correlated with the expression of *DilBCAT*1, -11, and -14 in all three cultivars, and negatively correlated with the *DilBCAT*9 in LDB and XC (*DilBCAT*9 expression in SX was about zero), and significantly negatively correlated with the *DilBCAT* 6 in LDB and XC (*DilBCAT*6 expression in SX was undetectable). However, the content of Leu showed a positive relationship with the expression of *DilBCAT*16 in all three varieties, and SX reached a very significant level.

## 4. Discussion

### 4.1. FAAs Profile of Longan Cultivars with Different Aroma Types during Fruit Development

The compositions and abundance of FAAs are important for human nutrition and health [32], and they can also affect the taste and aroma quality of foods [33,34]. Most of the previous studies only detected FAAs in longan mature fruit. For instance, Wang et al. [19] determined 17 FAAs in ‘Youtanben’ fresh fruit, with a total content of 9.3 g∙kg^−1^. Peng et al. [20] determined 18 FAAs in ‘Shixia’ and ‘Chuliang’ juice, with a total content of 5.0 g∙kg^−1^ and 4.8 g∙kg^−1^, respectively. Khan et al. [35] identified a total of 27 FAAs in dried longan pulp. Based on a widely targeted metabolomics approach, Wang et al. [6] had identified 706 metabolites (including ~98 FAAs and their derivatives) from fruit pulp, peel, and seed in two longan varieties i.e., ‘Shixia’ and ‘Chuliang’. Besides, Huang et al. [21] determined 18 kinds of FAAs in ‘Lidongben’ during the fruit development phase and tree hanging phase (3 stages each), the total content of the 18 FAAs ranged from 7.6 to 9.1 g∙kg^−1^, but the selected variety was a non-aromatic genotype. Therefore, over-arching differences in the FAAs metabolic profiles of longan cultivars with different aroma types during fruit development have not been investigated thoroughly until now.

In this study, based on the automatic amino acid analyzer, we detected and analyzed the dynamic changes of FAAs in the six developmental stages of three longan cultivars with different aroma types. 36 FAAs (including 17 protein amino acids and 19 amino acid metabolites) were detected in pulp, and the total content ranged from 2.6 to 9.1 g∙kg^−1^. Among these, Glu, Ala, Arg, g-ABA, Asp, Leu, Hypro, and Ser were the predominant FAAs (Figure 1, Table S1), which were consistent with previous studies, such as Huang et al. [21], Wang et al. [19], and Peng et al. [20]. In addition, the analysis showed that the aromatic variety (XC) tends to accumulate more Glu, Ala, Ser, and Leu than the non-aroma genotype (SX and LDB) during fruit growth (Figure 2C). Ala is formed by pyruvate and Glu under the action of alanine aminotransferase [31]. The Ala and Glu contents in the current study were significantly positively correlated in the three varieties (Figure 3H–J), suggesting that the accumulation of Glu during longan fruit development, especially in the rapid expansion period (S2–S4), could promote the formation of Ala. Meanwhile, the Ala and Leu contents were significantly positively correlated in LDB and XC and positively correlated in SX (r = 0.870) (Tables S4–S6), indicating that Ala was an important substrate for Leu synthesis in longan fruit.

### 4.2. Genes Involved in the Differential Accumulation of BCAAs in Pulp of Longan Cultivars with Different Aroma Types

While the process of neofunctionalization is characterized by gene duplication, both IPMS (2 copy) and IPMD (2 copy) in longan are low copy genes, which is consistent with some reported plants, such as 2 copy IPMS [36] and 3 copy IPMD [37,38] in Arabidopsis, 1 copy IPMS in banana [39], 4 copy IPMS in tomato [40], 2 copy IPMS [18] and 1 copy IPMD [17] in rice. In contrast, the BCAT genes in the longan and litchi genome were enriched, both presenting 16 copy, compared with the 7 copy and 6 copy BCAT genes observed in Arabidopsis [25] and tomato [26], respectively. The expression levels of *DilIPMS* and *DilIPMD* genes were relatively high (average FPKM~40), which were stable in different stages and varieties, while the distinctions of *DilBCAT* genes were sharp (Table S1). It is speculated that the low copy and relatively stable expression of IPMS and IPMD in the longan genome may be related to their functional conservation as rate-limiting enzyme genes of Leu biosynthesis. In addition, the duplication, expansion, and differential expression of BCAT are conducive to maintaining its functional diversity as a reversible catalytic enzyme gene in the metabolism of BCAAs.

The interface of BCAAs metabolism in plants lies with BCAT that catalyzes both the last anabolic step and the initial catabolic step. Previous analysis had indicated the gene expression of BCAT is related to its subcellular location [30,41], tissue specificity, developmental stage [25], etc. For example, Tomato encoded mitochondrial (mainly function as catabolism), chloroplast (mainly function as synthesis), cytosol, and vacuole located BCAT, respectively [26]. Our phylogenetic analysis showed that the 16 *DilBCATs* could be divided into four groups: group I and IV with high or medium expression levels, and group II and III with medium or low expression (Figure 3, Table S1). Among them, *DilBCAT*11 likely functions as a major enzyme for BCAA synthesis (especially Leu), since it has the highest and nearly stable expression at all stages of fruit development of three varieties. While *DilBCAT*15 may function as catabolism, its gene expression gradually decreased with fruit maturity, and there was a significant negative correlation with Leu content in SX and XC. The transcript levels of *DilBCAT*1, -6, and -9 in XC were significantly higher than those in SX and LDB and were negatively correlated with Leu content. While *DilBCAT*16 was just the opposite, suggesting that these four genes played an important role in the difference of BCAA content accumulation among varieties. Further functional identification of these *DilBCAT* genes is necessary, such as yeast complementation analysis, enzyme-substrate specificity, tissue expression specificity, subcellular localization, transgenic verification, etc.

### 4.3. BCAAs-Derived Volatile Compounds in Longan Fruit 

Branched-chain esters, derived from BCAAs, are important contributors to the fruit aroma of several horticultural crops [13,14,15,16]. Although ~100 aroma components have been detected in longan fruit [9], little attention has been paid to the branched-chain volatiles. The addition of Ile and Leu to longan juice during fermentation could significantly increase the formation of 2-methyl-1-butanol and 3-methyl butanol and their corresponding acetate esters, respectively, and then affected the flavor of longan wine [42,43]. Besides, Zhang detected diisobutyl adipate from the juice of Chuliang variety by the distillation extraction method [44], which was an ester formed by 2-methyl-propanol produced from Val (Figure 3A). By using headspace-solid phase microextraction combined with GC/MS, we also detected 3-Methylbutyraldehyde and 3-methyl-butanol, which were derivatives of Leu, in the early developing fruit of XC (data to be published). Therefore, we speculated that the branched-chain volatiles may play a specific role in the aroma quality of longan fruit. For these targeted aroma compounds, it is necessary to further carry out mass spectrometry standard validation [45], sensory evaluation [45], exogenous amino or α-keto acids promotion of synthesis [46], carbon isotope analysis of biochemical origins [16,46], and then finally clarify the mechanism of free BCAAs involved in the formation of longan fruit aroma.

## 5. Conclusions

The results of the current study suggest that Glu, Ala, Arg, GABA, Asp, Leu, Hypro, and Ser were the predominant FAAs in longan pulp. During the period of rapid fruit expansion, the contents of Ala and Leu in aromatic variety (XC) were significantly higher than those in non-aromatic varieties (SX and LDB). Correlation analysis showed that *DilBCAT1*, -6, -9, and -16 may be responsible for the synthesis or degradation of Leu among studied varieties. This study provides new insights for elucidation of the metabolic mechanism of FAAs and the formation of aroma quality in longan fruit. 

## Data Availability

Not applicable.

## Supplementary Materials

The following are available online at https://www.mdpi.com/article/10.3390/biology10080807/s1. Figure S1: PLS-DA significance diagnostic for FAAs of three longan cultivars at different maturity stages, Table S1: The list of 36 free amino acid detected in longan pulp, Table S2: Information and expression pattern about the amino acid metabolic genes family in Longan, Table S3: Information about the BCAT genes in Litchi, Table S4: Correlation analysis of FAAs content and BCAAs synthesis-related genes expression in SX, Table S5: Correlation analysis of FAAs content and BCAAs synthesis-related genes expression in LDB, Table S6: Correlation analysis of FAAs content and BCAAs synthesis-related genes expression in XC.
