# Supplementary material for: Free Amino Acids Profile and Expression Analysis of Core Genes Involved in Branched-Chain Amino Acids Metabolism during Fruit Development of Longan (Dimocarpus longan Lour.) Cultivars with Different Aroma Types
Source: Biology (Basel). 2021 Aug 20;10(8):807. doi: 10.3390/biology10080807 (PMC8389590; doi:10.3390/biology10080807)
Supplement: Supplementary file 1 [file biology-10-00807-s001.zip › biology-1297068-supplementary/Figure S1.pdf]

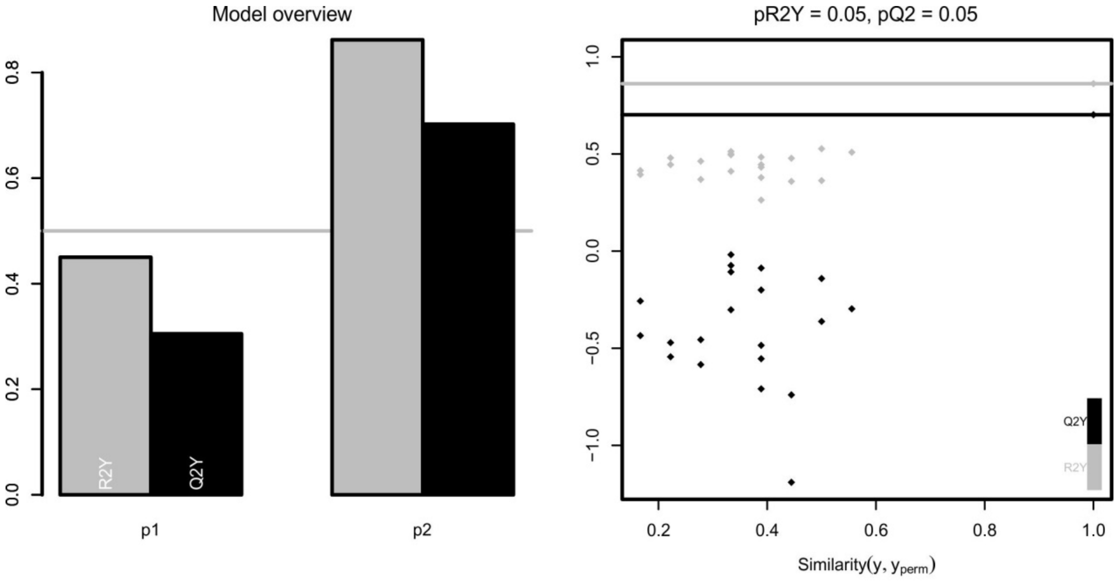

Figure S1 PLS-DA significance diagnostic for FAAs of three longan clutivars at different maturation stages.
